# Twisting Ends of Arteries

**Published:** 1869-02

**Authors:** 


TWISTING ENDS OF ARTERIES.—The practice of twisting the divided ends of the arteries, instead of applying ligatures, has lately been tried by Professor Humphry after all his operations, in Addenbrooke's Hospital, Cambridge, and with good result. There has been no subsequent bleeding in any case; and the wounds have, he thinks, on the whole, healed better than they would have done with the ligatures. The popliteal is among the arteries which have been thus secured, and the femoral, in two instances, after amputation in the thigh.—*Lancet.*

